# A ventral stream-prefrontal cortex processing cascade enables working memory gating dynamics

**DOI:** 10.1038/s42003-022-04048-7

**Published:** 2022-10-12

**Authors:** Shijing Yu, Sarah Rempel, Negin Gholamipourbarogh, Christian Beste

**Affiliations:** Cognitive Neurophysiology, Department of Child and Adolescent Psychiatry, Faculty of Medicine, TU Dresden, Dresden, Germany

**Keywords:** Cognitive control, Cognitive neuroscience

## Abstract

The representation of incoming information, goals and the flexible processing of these are required for cognitive control. Efficient mechanisms are needed to decide when it is important that novel information enters working memory (WM) and when these WM ‘gates’ have to be closed. Compared to neural foundations of maintaining information in WM, considerably less is known about what neural mechanisms underlie the representational dynamics during WM gating. Using different EEG analysis methods, we trace the path of mental representations along the human cortex during WM gate opening and closing. We show temporally nested representational dynamics during WM gate opening and closing depending on multiple independent neural activity profiles. These activity profiles are attributable to a ventral stream-prefrontal cortex processing cascade. The representational dynamics start in the ventral stream during WM gate opening and WM gate closing before prefrontal cortical regions are modulated. A regional specific activity profile is shown within the prefrontal cortex depending on whether WM gates are opened or closed, matching overarching concepts of prefrontal cortex functions. The study closes an essential conceptual gap detailing the neural dynamics underlying how mental representations drive the WM gate to open or close to enable WM functions such as updating and maintenance.

## Abstract (web summary)

Analysis of representational dynamics during working memory gating suggests that distinct opening and closing gating activity profiles are attributable to a ventral stream-prefrontal cortex processing cascade.

## Introduction

For decades, executive functions and cognitive control processes have been subject to neuroscience research and refer to a set of functions necessary for goal-directed behavior. According to current taxonomies, there are three major classes of executive functions—working memory processes, interference control, and cognitive flexibility processes^1^. Each of these entities has been subject to intense research, and the neurophysiological processes (e.g., as measured using EEG) have been intensively investigated.

Regarding working memory (WM) functions, considerable research has explained the processes involved in WM maintenance (i.e., how information is kept in WM). Substantially less research is evident regarding the neurophysiological processes involved in WM gating processes—how information enters WM and how this process is controlled. Computational models suggest that WM content is regulated by an input-gating mechanism^2,3^. When the gate is open, the updating of WM content is possible; when the gate is closed, WM content is relatively stable and resistant to interfering information. The decision when to open or close the working memory gate depends on the utility of the information presented^4^. The detection of relevant information leads to the opening of the gate^5^. As opposed to this, when goal-unrelated information is detected and likely disrupting ongoing behavior, the gate is closed^6^. These gate opening and closing dynamics are central for goal-directed behavior, which depends on the arbitration between different states of cognitive persistence and flexibility^7–9^. Despite this importance, only recently, the behavioral and neurophysiological mechanisms underlying WM gate opening and closing processes have come more into focus^3,10–12^. Rac-Lubashevsky and Kessler^13^ proposed a reference-back paradigm to manipulate and measure WM gating processes (see “Methods” for details). There, WM gate opening is indicated by a switch from WM maintenance to updating, while WM gate closing is indicated by a switch from updating to maintenance. Previous studies using this paradigm consistently reported higher reaction time (RT) cost and lower accuracy cost in gate closing than in gate opening^3,11–13^. According to the prefrontal cortex basal ganglia working memory (PBWM) model, the default mode of the WM gate is the closed state (i.e., maintenance state). The low accuracy cost in gate closing is intuitive as the closing process is a switch from updating (high demanding status) to maintenance (low demanding default status). The high RT cost in gate closing can be explained by the previous finding that switching from a more challenging task to an easier task takes longer than the other way around^14^. Neurophysiological evidence also suggests different patterns between WM gate opening and closing processes^4,10,11^. These differences are likely to be driven by the information representing a change from the goal of the immediate past to the goal evident in the current situation (trial). Specifically, when the WM gate is closed, i.e., the goal is maintained, an upcoming stimulus requiring updating will trigger the WM gate to open. On the contrary, when the WM gate is open, an upcoming stimulus requiring maintenance will trigger the gate to close. Therefore, the mental representations driving the WM gating processes are not simply stimulus-related but also tightly related to the WM gate states (open or closed). The representations examined in this study thus contain comparative information about the immediate past relative to the current stimulus information and the rule to be applied. These complex representations are central for cognitive framings of working memory processes which further guide other cognitive processes (e.g., cognitive control and goal-directed behavior)^15–17^. While previous work mostly studied representations when information has already entered WM (i.e., is actively maintained in WM buffers), the conceptually relevant question regarding the regulation of representations’ access to WM has not yet been answered: How are representations affected during WM gate opening/closing and what neurophysiological processes and neuroanatomical structures are implied in this process?

The advent of sophisticated multivariate pattern analysis (MVPA) methods applied to neurophysiological (EEG) data likely answers this conceptually relevant question that is also informative for cognitive science frameworks focusing on WM gating processes. MVPA, also known as multi-voxel pattern analysis, can decode the difference between experimental conditions based on the observed neural patterns^18,19^ and is suitable to answer questions on how mental representations are handled during cognitive operations^18–27^. Especially when applying temporal generalization MVPA suitable to examine when and for how long representations are activated, it is possible to distinguish activity patterns in the temporal generalization matrix with different conceptual implications^23,24^. The current study uses temporal generalization MVPA in a well-established experimental approach^3,11,12^ to capture the representational dynamics of WM gate opening and closing processes on a neurophysiological (EEG) level. The representational dynamics is only possible to capture using MVPA^23^ because more traditional event-related potentials (ERPs) cannot capture the full information being evident in the EEG signal (i.e., its multivariate nature)^28^, and no generalization of processes captured can be tested across time and content^23^, which is, however, necessary when being interested in cognitive constructs (e.g., working memory) where the temporal stability of a representation plays an essential role. However, when considering decoding the complex representations during WM gate opening and gate closing processes, it is essential to note that WM dynamics reflect distributed processes in multiple regions of the temporal, parietal, and prefrontal cortices^2,11,29^. These spatial properties of the EEG signal thus need to be considered in detail. This, again, has repercussions on the choice of neurophysiological (EEG) data analysis methods because the EEG, due to the volume conduction effect, reflects a mixture of signals from various brain regions^30,31^. The best way to approach this is to perform a blind source separation method—independent component analysis (ICA)^30^. Considering that WM processes are distributed across cortical structures^29^, there may be different neurally independent components of processes also involved in WM gate opening and closing and its representational dynamics. It is possible that dissociable spatial activity profiles in the EEG reveal distinct temporal representational dynamics. To capture this, it is necessary to combine ICA and MVPA (i.e., to apply MVPA on isolated independent spatial activity profiles constituting the EEG).

Importantly, this combination of methods can still not inform about the functional neuroanatomical structures associated with this dynamic, since scalp recorded EEG data is not directly reflect the generating functional neuroanatomical structure due to the inverse problem^32^. To solve this, source localization methods are necessary. For time periods in which evident decoding of the representational content in an independent component during WM gate opening and closing processes was possible, we apply source localization methods to delineate which functional neuroanatomical structures are associated with the processes^24^. Likely, temporal, parietal, and prefrontal regions are associated with these dynamics as these regions are also involved in maintaining WM content^2,29^. However, it is elusive whether these cortical regions reveal a specific temporal cascade in the pattern of activity modulations while handling the WM representational content during gate opening and closing processes.

To summarize, the current study aims to combine EEG signal decomposition (ICA), temporal generalization multivariate pattern analysis (MVPA), and source localization to provide a comprehensive picture of the neurophysiological mechanisms underlying the temporal stability of WM representations during WM gate opening and closing. This is not only relevant to better understand the neural processes of WM gating but will also inform cognitive frameworks on working memory gating processes because these frameworks do currently not make explicit assumptions about the temporal dynamics representations during WM gating.

## Results and discussion

### Behavioral data—replication of previous studies

**Fig. 1:**
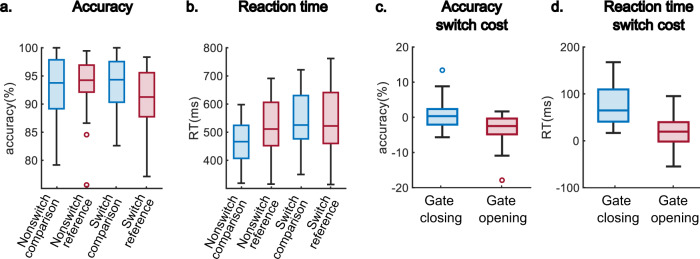
Behavioral results for gate opening and gate closing. Behavioral results derive from *N* = 33 participants. Plots **a** and **b** reveal the accuracy and reaction time (RT) in each condition, respectively. Plots **c** and **d** show the switching costs of accuracy and RT. For each box, the central line indicates the median value, the bottom edge of the box indicates the 25th percentiles, and the top edge indicates the 75th, and each dot represents an outlier identified by Matlab’s ‘boxchart’ function.

The behavioral performance of four conditions (i.e., switch_reference, nonswitch_reference, switch_comparison, and nonswitch_comparison) is shown in Fig. 1a and b. The four conditions represent different trial types defined by two factors. One factor differentiates trials/stimulus between reference and comparison that reference stimulus provide the crucial information for upcoming tasks, hence should be maintained in WM, oppositely, comparison stimulus contains no reference information and should be removed from WM after the task is completed. The other factor indicates a (non)switch process between reference and comparison trials. With a switch process, the WM gate changes its states from open to closed when switching from comparison to reference trials, and from open to closed when switching from reference to comparison trials (see “Methods” for details). Figure 1c and d illustrates the switching costs during gate opening and closing processes using accuracy and RT. The WM gating processes were calculated as

1 $${Gate\; opening}={switch}{{\_}}{reference}-{nonswitch}{{\_}}{reference}$$

2 $${Gate\; closing}={switch}{{\_}}{comparison}-{nonswitch}{{\_}}{comparison}$$

Namely, the behavioral indices of gate opening and closing were calculated as the switching cost of reference trials and comparison trials. That is, the reference trials required participants to update the reference information; hence a switch_reference stimulus triggered the gate opening, while a nonswitch_reference stimulus reflected the open status of the gate. Similarly, the comparison trials required participants to maintain the information. Therefore, the switch_comparison stimulus triggered the gate closing (from updating to maintenance), while a nonswitch_comparison trial represents the closed status of the gate. In both gating computations, the nonswitch trials reflect a baseline of the gate status.

Following the two formulas (Eqs. 1 and 2), the switch cost of the accuracy of the gate opening (−3.17 ± 3.99%) was significant larger compared to the gate closing (0.61 ± 3.94%; t(32) = −3.22, *p* = 0.003, Cohen’s *d* = −0.56). The switch cost of the RT during the gate opening (22.80 ± 31.72 ms) were significant lower than it during gate closing (74.16 ± 40.35 ms; t(32) = −6.53, *p* < 0.001, Cohen’s *d* = −1.14). These behavioral data replicate previous findings using the same task^3,10–12^ showing that the WM gate opening process is associated with a higher cost of accuracy but lower cost of reaction time compared with the WM gate closing process. According to Schneider and Anderson^14^, the RT cost of switching from an easier task to a more challenging task is larger than the other way around due to impaired performance after a difficult trial. This, together with the lower cost of accuracy in WM gate closing, suggests that WM gate closing is easier than WM gate opening. Gate opening enables new, behaviorally relevant information for goal-directed behavior^5^. Gate closing is necessary to shield ongoing behavior from distracting information^6^. Gate opening and closing processes are thus relevant for the arbitration between cognitive persistence and flexibility states, necessary to achieve longer-range behavioral goals^7–9^. This arbitration is reflected in complex neurophysiological dynamics of multiple independent neural activity profiles as outlined below.

### Multiple independent neural activity profiles reflect WM gate opening and closing

**Fig. 2:**
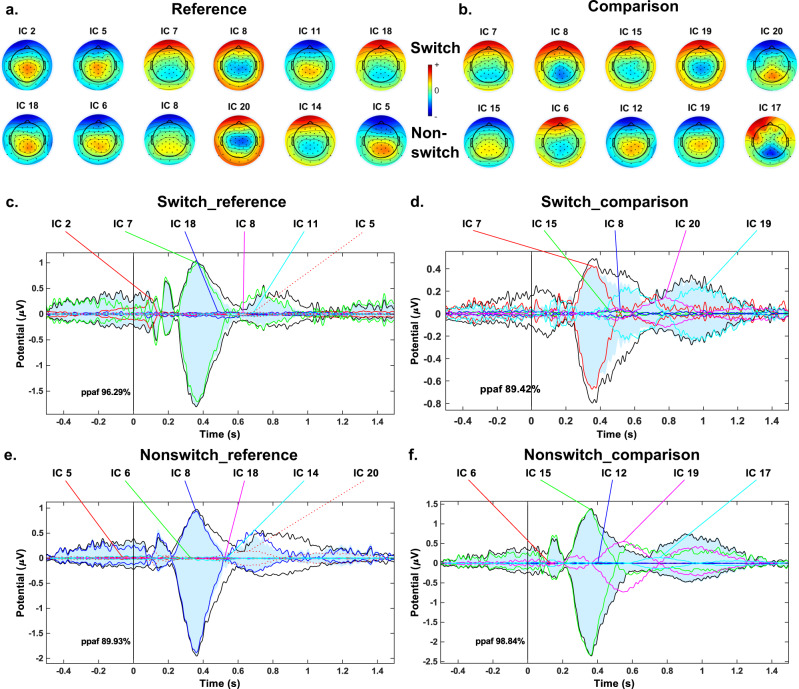
Similar independent component (IC) pairs and corresponding ERPs for gate opening and closing conditions. Plots **a** and **b** show similar IC pairs in gate opening and closing conditions. Each column shows one pair of similar ICs, with the first row representing switch trials and the second-row representing nonswitch trials. Arbitrary units are applied to the scale of the IC topographies. Plots **c**–**f** present the component ERPs corresponding to each ICs from plots **a** and **b**.

To account for a complex and possibly spatially independent neural dynamics during working memory gate opening and closing, we used group independent component analysis (Group-ICA) to decompose the EEG data from the four conditions (i.e., switch_reference, nonswitch_reference, switch_comparison, nonswitch_comparison). The algorithmic and statistical reliability of group independent components (ICs) were estimated using the ICASSO method^33^. Details on these procedures are provided in the “Methods” section. The ICASSO results showed that the average stability (*I*_q_) of the IC components was 0.92 ± 0.07 for the switch_reference condition, 0.98 ± 0.01 for the nonswitch_reference condition, 0.96 ± 0.03 for the switch_comparison condition, and 0.97 ± 0.01 for the nonswitch_comparison condition. Supplementary Fig. 1 shows the 2D visualization of components similarity in ICASSO algorithm for all four conditions. Also, the result of the R-index calculation that the quality of the clustering demonstrated was higher with 20 clusters (equal to the number of components) than with fewer for all conditions. As shown in Supplementary Fig. 2, the 20 clusters that show the best clustering quality had the lowest values of R-indices in all four conditions (i.e., switch_reference, nonswitch_reference, switch_comparison, nonswitch_comparison). After extracting reliable group components for gate opening and gate closing conditions using ICASSO, we applied CORRMAP as a clustering technique to find spatially similar components between different task conditions of gate closing and gate opening, because the components of different conditions could occur with arbitrary scales and orders and it is necessary to match components between conditions. The CORRMAP procedure extracted 7 pairs of homogeneous ICs between switch_reference trials and nonswitch_reference trials for the gate opening condition (see Table 1) and 5 pairs of homogeneous ICs between switch_comparison trials and nonswitch_comparison trials for the gate closing condition (see Table 2). The stability of each IC calculated from the ICASSO, and the similarity represented by the correlation of ICA inverse weights of each pair (calculated by CORRMAP), are also presented in Tables 1 and 2. The stabilities of most ICs were relatively high except for the 20th IC in the switch-reference condition, which was at 0.65 (all the rest *I*_q_ ≥ 0.88). Due to that, we excluded the 7th pair of ICs in gate opening conditions (i.e., IC 20 for the switch_reference condition and IC 19 for the nonswitch_reference condition) from further analysis. The similarities of the remaining IC pairs were also relatively high (all between 0.92 and 0.99). Figure 2a and b shows the topographies of the remaining ICs. The back-projected activity (i.e., averaged from all channels) for each IC is shown in Fig. 2c–f. The sum of percentage of power accounted for (ppaf) of the activities back-projected from the above-selected ICs was also calculated and results showed in each condition, it was higher than 89%. It suggests that the selected ICs were sufficient and likely to reconstruct the original EEG signals. The ppaf of the back-projected activities of individual ICs are presented in Supplementary Tables 1 and 2. Since these results demonstrate that there multiple independent components constituting activity underlying WM gate opening and closing (and not only one component for each) the findings suggest that WM gate opening and closing is not constituted by a single neural process. Rather, multiple neural processes are important, which also reveal a complex (nested) temporal dynamics as show below. Previous study of our group also revealed that by using Group-ICA and ICASSO, the EEG signal can be decomposed into components carrying different informational aspects or processing codes relevant for perception-action integration^34^.

**Table 1:** Independent component (IC) stability and IC pair similarity in the gate opening condition.

Pair index		1	2	3	4	5	6	7
IC index (*I*_q_)	Switch reference	IC 2 (0.92)	IC 5 (0.96)	IC 7 (0.95)	IC 8 (0.96)	IC 11 (0.94)	IC 18 (0.90)	IC 20 (0.65)
	Nonswitch reference	IC 18 (0.97)	IC 6 (0.97)	IC 8 (0.98)	IC 20 (0.98)	IC 14 (0.97)	IC 5 (0.98)	IC 19 (0.98)
Similarity		0.95	0.95	0.99	0.92	0.98	0.95	0.96

**Table 2:** Independent component (IC) stability and IC pair similarity in the gate closing condition.

Pair index		1	2	3	4	5
IC index (*I*_q_)	Switch comparison	IC 7 (0.98)	IC 8 (0.97)	IC 15 (0.96)	IC 19 (0.88)	IC 20 (0.93)
	Nonswitch comparison	IC 15 (0.98)	IC 6 (0.97)	IC 12 (0.97)	IC 19 (0.98)	IC 17 (0.97)
Similarity		0.96	0.94	0.95	0.97	0.96

### Nested representational dynamics during WM gating

**Fig. 3:**
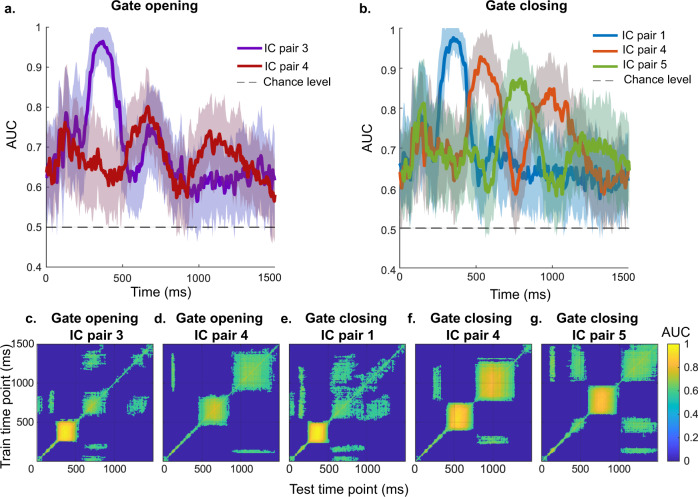
MVPA results for selected independent component (IC) pairs of gating conditions. Plots **a** and **b** show the binary classification performance of gate opening and closing conditions separately. The shaded error bars represent standard deviation. Plots **c**–**g** illustrate the temporary generalization of each IC pair. Only time points with significant (*p* < 0.05) classification performance computed by cluster-based permutation test with *N* = 33 samples are presented in color.

To examine the representational dynamics of the isolated activity in detailed, we applied MVPA on each isolated and back-projected IC pairs reflecting gate opening and closing. Two analyses were executed at the single-subject level for each pair: a binary classification across time to identify the time points showing different patterns between IC pairs and a temporal generalization analysis to characterize the temporal dynamics of the representational content at the component ERP. The binary classification performance across time and the temporal generalization matrix are shown in Fig. 3 and Supplementary Fig. 3. The binary classification performance of all IC pairs was significantly higher than the chance level (*p* < 0.05). However, evident area under the curve (AUC) peaks of binary classification and temporal generalization clusters representing significant AUC values (AUC > 0.5, *p* < 0.05) were only detected in some IC pairs, i.e., IC pairs 3 and 4 in the gate opening condition, and IC pairs 1, 4, and 5 in the gate closing condition. Therefore, we only present the MVPA performance of the abovementioned IC pairs in Fig. 3. Please refer to Supplementary Fig. 3 for the MVPA performance of the rest IC pairs.

For the IC pair 3 of the gate opening conditions, the AUC curve of the binary classification across time (Fig. 3a) revealed one peak of 0.96 at 371 ms after stimulus onset. The temporal generalization of IC pair 3 showed a cluster of significant above-chance off-diagonal activity between 250 and 500 ms (Fig. 3c). Another smaller peak of 0.76 was also observed around 668 ms after stimulus onset, and the corresponding temporal generalization revealed a small cluster of significant off-diagonal activity between 600 and 800 ms. In the binary classification step, two peaks were observed for the IC pair 4 of gate opening conditions. The first one reached an AUC value of 0.80 at 664 ms, and the corresponding temporal generalization cluster also centralized around 650 ms after stimulus onset with an off-diagonal activity of ~300 ms (Fig. 3d). The second peak was slightly lower but reached an AUC value of 0.74, which was evident at 1070 ms after stimulus onset. The second peak’s corresponding temporal generalization cluster (i.e., off-diagonal activity) was observed around 900 to 1400 ms after stimulus onset.

For gate closing IC pairs (Fig. 3b), the AUC curve of the binary classification of IC pair 1 showed a peak of 0.98 at 352 ms after stimulus presentation and an off-diagonal activity (temporal generalization) of ~250 ms. For IC pair 4, two peaks of the AUC curve were detected in the binary classification, corresponding to the two clusters showing significant high performance in the temporal generalization. The first peak reached 0.92 at 527 ms after stimulus onset, and the corresponding temporal generalization cluster expanded from around 400 ms to around 750 ms after stimulus onset. The second peak reached 0.85 at 980 ms after stimulus onset, and the corresponding temporal generalization cluster spread from 800 ms to 1300 ms. The AUC performance of the binary classification using the IC pair 5 data in the gate closing also reached a peak of 0.97 at 785 ms after stimulus onset. The temporal generalization showed a cluster of significant classification performance (AUC > 0.5, *p* < 0.05) around 600 to 1000 ms after stimulus onset.

As abovementioned and can be seen in Fig. 3a and b, the different independent components show an alternating pattern of activity—especially during the WM gate closing dynamic (Fig. 3b). Thus, there is a pattern of recurrent and nested higher and lower activation in the individual ICs and the associated representational dynamic (Fig. 3c–g). The two independent spatial neural activity profiles occurring ~400 ms after presented stimulus information explain significant neural activity proportions during WM gate opening. During WM gate closing, three independent spatial neural activity profiles, again occurring ~400 ms after stimulus information, accounted for large proportions of the neural activity. This shows that WM gate opening and closing processes revealed a similar temporal dynamic. However, gate closing processes depend on slightly more complex neural dynamics than gate opening processes. This aspect is, at present, not covered in the conceptual account of WM gate opening and closing. The interpretation is corroborated by the more nested temporal pattern of activations of the representations during WM gate closing than gate opening. During WM gate closing, the identified ICs showed a systematic temporal pattern whereby the peak in classification accuracy based on one IC was followed by a peak in classification accuracy based on another IC. In this respect, gate closing reveals a dynamic of an intermittent activation of representational contents, which could be assigned to independent neuronal activity patterns. This pattern was less evident during WM gate opening processes (see AUC curve in Fig. 3a).

### A ventral stream-prefrontal cortex cascade in nested WM gating representational dynamics

**Fig. 4:**
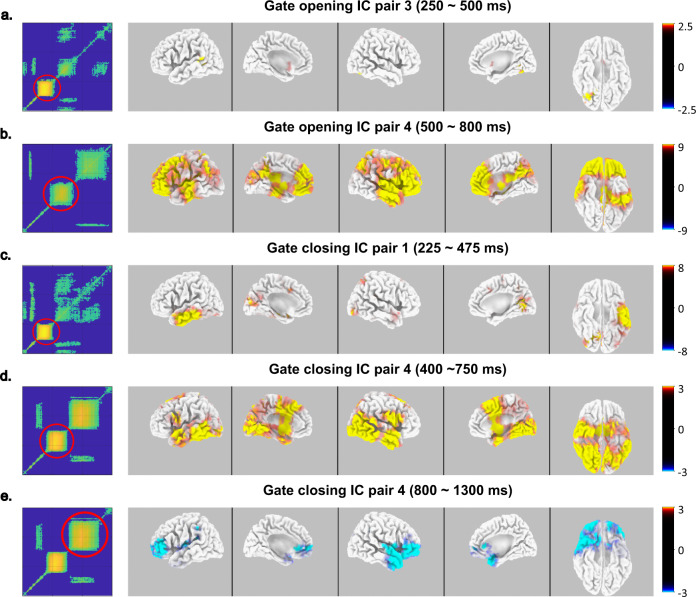
Source localization of working memory gating dynamics. The title indicates the independent component (IC) pair and the time window selected for sLORETA analysis for each plot (**a**–**e**). The temporal generalization result of this IC pair is presented on the left in each plot from (**a**) to (**e**) with a red circle marking the cluster utilized for sLORETA. The source of the working memory gating dynamics is illustrated from five orientations (from left to right: lateral-left, medial-left, lateral-right, medial-right, and bottom) presented on the right in each plot from (**a**) to (**e**). The color bar represents the critical *t*-values using *N* = 33 samples. The color key on the right of each plot from (**a**) to (**e**) denotes *t*-values.

Source localization using the sLORETA software package^35^ was used to examine which functional neuroanatomical structures are associated with the representational dynamics captured in the different independent components and their temporal modulation, as outlined in the above section and Fig. 3. The source localization analysis tracks how brain regions from the ventral stream pathway to the prefrontal cortex are activated during WM gate opening. The results of the source localization analysis are shown in Fig. 4.

During working memory gate opening (Fig. 4a), representational content reflected between 250 and 500 ms in IC pair 3 was associated with significant activity modulations in the ventral stream encompassing the lingual gyrus (BA19) and the superior temporal gyrus (BA22). No significant sources could be obtained for the off-diagonal activity between 600 and 800 ms. For the representational dynamics during gate opening processes reflected by IC pair 4 (Fig. 4b) centralized around 650 ms after stimulus onset, significant bilateral source activity was found extending large parts of the anterior portions of the inferior, middle, and superior temporal cortices (BA20, BA21, BA22). Moreover, large parts of the inferior and dorsolateral prefrontal cortex revealed bilateral activity modulations. These modulations included the inferior frontal gyri (BA47) and the middle and superior frontal gyri, including frontopolar cortices (BA9, BA10, BA11, BA47). In addition, the right inferior parietal regions (BA40) revealed activity modulations. For the other representational dynamics during gate opening processes reflected by IC pair 4 around 900 to 1400 ms after stimulus onset, no significant source was detected by sLORETA analysis. The representational dynamics during WM gate opening processes thus start in the ventral stream and extends to frontal cortical regions. Theoretical approaches to prefrontal cortex functioning indicate that frontopolar regions play a role in branching control^36–38^, enabling them to maintain a state/information that may be useful in the future and revert to a pending task or episode following the completion of an ongoing one^36^. Such branching processes are assumed to precede processes guiding action selection^36^. Considering that the decision to open a WM gate depends on the estimated utility of information^4^, the involvement of cortical regions for which other theoretical concepts have stated are central to estimating whether a response option is useful seems reasonable. The current findings thus provide a conceptual link between WM gating processes and an information approach to prefrontal cortex function^36^ in that cognitive branching is increased during working memory gating, opening representational dynamics. Interestingly, the frontopolar region is closely connected to regions in the temporal cortex^37,39,40^, shown to be associated with the representational content processing before the representations during WM gate opening and closing become associated with prefrontal regions. It may be speculated that such structural connections underlie the observed ventral stream-prefrontal cortex cascade in WM gating representational dynamics. Notably, frontopolar regions have not been reported to be associated with WM maintenance^29^. Thus, the dynamics of WM gating processes cannot be directly inferred from knowledge about the mechanisms of WM maintenance.

**Fig. 5:**
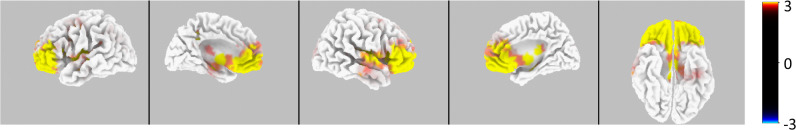
Source difference of working memory gating dynamics between 400 and 750 ms. The source difference was computed using gate opening independent component (IC) pair 4 and gate closing IC pair 4. The source difference of the working memory gating dynamics is illustrated from five orientations (from left to right: lateral-left, medial-left, lateral-right, medial-right, and bottom). The color bar represents the critical *t*-values using *N* = 33 samples. The color key on the right denotes *t*-values.

During working memory gate closing (Fig. 4c–e), representational content reflected by IC pair 1 at 352 ms after stimulus presentation with a temporal generalization of ~250 ms was mostly associated with activity modulations in left-sided anterior temporal cortices (BA20, BA21). For IC pair 4, the first temporal generalization from around 400 ms to around 750 ms after stimulus onset was associated with bilateral activity modulations in temporal cortices (BA20, BA21, BA22) and the lingual gyrus (BA19) as well as the precuneus. Moreover, bilateral medial and superior frontal regions revealed activity modulations (BA6, BA8, BA9, BA32). The second temporal generalization cluster between 800 ms and 1300 ms after stimulus presentation was associated with activity modulations in frontopolar and orbito-frontal cortices (BA10, BA11, BA46, BA47). For IC pair 5, no significant activity clusters in the sLORETA analysis were found. The representational dynamics during WM gate closing start in the ventral stream and extends to frontal cortical regions, similar to WM gate opening processes. During WM gate closing, and as opposed to WM gate opening, the representational dynamics were associated with bilateral medial and superior frontal regions revealed activity modulations (BA6, BA8, BA9, BA32) between 400 and 750 ms after stimulus presentation. Gate closing is initiated when distracting or conflicting information is detected^6^. Medial and superior prefrontal regions have consistently been associated with conflict monitoring processes^41–43^. From that perspective, it is reasonable that representational dynamics during WM gate closing are associated with medial and superior frontal cortex activity. This conflict-related process is not evident during the representational dynamics associated with WM gate opening (see above). Crucially, frontopolar and orbitofrontal regions were also modulated in the representational dynamics during WM gate closing processes, but later (i.e., between 800 and 1300 ms) and the direction of activity as revealed by the sLORETA contrast was negative. The data suggest that cognitive branching^36,37,44^ is downregulated during the gate closing representational dynamics, probably a consequence of conflict monitoring. This is substantiated by the finding that the same extracted independent component (IC) was associated with representational dynamics in earlier time conflict-monitoring-related windows (i.e., between 400 and 750 ms) and later time windows (i.e., between 800 and 1300 ms), revealing a decrease of activity in brain regions thought to be involved in cognitive branching. From the above, it appears that WM gate opening and closing processes differ in their involvement of prefrontal cortical areas. During WM gate opening, frontopolar, orbito-frontal, and dorsolateral prefrontal regions were activated (BA9, BA10, BA11, BA47). This was not the case during WM gate closing. By the source localization analysis directly contrasting WM gate opening and closing processes for the extracted independent components revealing temporal stability of the representational content between 400 and 750 ms after stimulus presentation (see Fig. 5), it is shown that frontopolar, orbito-frontal, and dorsolateral prefrontal regions (BA9, BA10, BA11, BA47) and regions in the medial frontal cortex encompassing the anterior cingulate cortex (BA24, BA32) were differentially activated between gate opening and closing.

Despite differences between working memory gate opening and closing discussed above, there are also commonalities between both processes. During gate opening and closing, representational content is first modulated between 250 and 500 ms, and the represented content is stable for ~250 ms. Of note, ventral stream cortical areas (lingual gyrus (BA19), superior temporal gyrus (BA22), and anterior temporal cortices (BA20, BA21) were associated with this dynamic in WM gate opening and closing. The ventral stream pathway processes what kind of information is coded in the presented stimuli^45,46^. During WM gating, stimulus-related content is thus evident for 250 ms in ventral stream pathways. Moreover, the regions identified are also modulated during WM maintenance^16,29^. During WM gate closing, the shielding process of WM content is also initiated in ventral stream pathways. The decision when to open or close the working memory gate depends on the utility of the information presented^4^. This utility estimation depends on what stimulus is detected and how it differs from the reference stored in WM. Therefore, it seems reasonable that WM gate opening and closing processes start within the ventral stream before other cortical regions are involved in WM gate opening and closing representation dynamics.

Taken together, using different EEG analysis methods, we traced the path of mental representations along the human cortex during WM gate opening and closing. Working memory gate opening and closing revealed a temporally nested representational dynamics depending on multiple independent neural activity profiles. These activity profiles are attributable to a ventral stream-prefrontal cortex processing cascade. The representational dynamics start in the ventral stream during WM gate opening and WM gate closing before prefrontal cortical regions are modulated. A regional-specific activity profile is shown within the prefrontal cortex depending on whether WM gates are opened or closed, matching overarching concepts of prefrontal cortex functions. The study closes an essential conceptual gap detailing the neural dynamics underlying how mental representations drive the WM gate to open or close to enable WM functions such as updating and maintenance

## Methods

### Participants

The study runs a quantitative, within-subject manipulation of experimental conditions. Data of Rempel et al.^11^ (*N* = 24 student individuals) and a newly-collected dataset (*N* = 18 student individuals) were chosen for this study (*N* = 42 individuals in total; 13 males, mean age 25.62 ± 5, all right-handers). To ensure a high quality of the group independent component analysis (ICA), we excluded *N* = 9 participants with an insufficient trial number (<50) for each condition after EEG segmentation. The final sample comprised *N* = 33 participants (9 males with a mean age of 25.77 ± 5.01). There was no randomization due to the within-subject design. The sample is comparable to other studies examining representational dynamics of neurophysiological activity using MVPA^25,26^. All participants had normal or corrected-to-normal vision, normal hearing capabilities, and no neurological and psychiatric disorders history. Participants of the newly collected data performed two other paradigms on the day of the experiment unrelated to the current study. The IRB of the TU Dresden approved the studies. Written informed consent was obtained from all individuals. All procedures followed the Declaration of Helsinki.

### Task

**Fig. 6:**
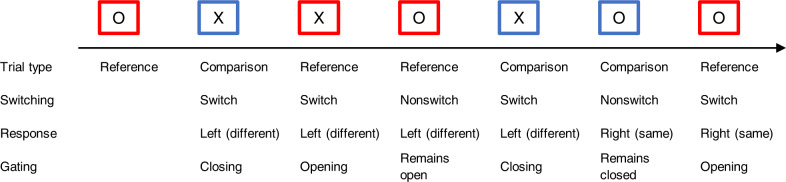
An example of the reference-back task. The arrow indicates the stimulus presentation order.

Rac-Lubashevsky and Kessler^3^ developed the Reference Back Task to investigate WM gating mechanisms. The task further develops the classical N-back task, which measures and manipulates WM gating processes. In this task, letters are presented, and participants are asked to decide whether the letter is identical to the letter presented N trials earlier. For this purpose, letters are displayed in blue or red frames (see Fig. 6). Only the letters X and O are used. Unlike in the N-back task, the further development by Rac-Lubashevsky and Kessler^3^ requires deciding whether each letter is identical to the letter that was last presented in a red frame. If a letter is presented in a blue frame, participants have to compare the letter only with the letter last presented in a red frame and not update WM.

In contrast, for letters presented in a red frame, participants must compare the letter with the letter last presented in a red frame and update WM. The presented letter then becomes the new reference. Trials with a red frame are referred to as reference trials, and trials with a blue frame are defined as comparison trials. Further aspects of a trial are relevant to investigating different gating mechanisms—a switch from a reference to a comparison trial or vice versa. If, on the other hand, two trials of the same kind follow each other (i.e., reference to reference or comparison to comparison), it is defined as a nonswitch trial. These two aspects (trial type, switching) result in four possible conditions: switch_reference, nonswitch_reference, switch_comparison, and nonswitch_comparison. In the task, ‘N’ (i.e., the distance to the referenced WM item and the distance to the referenced WM item) is not controlled. However, this does not confound the computation of gating behavior: The reference-back task component in the paradigm is, simply speaking, used to compare the current stimulus with the previous red-framed stimulus. As explained by Rac-Luashevsky et al.^13^, ‘… this feature makes the reference-back similar to a 1-back task, since they both require maintaining only one item in WM’. According to Watter et al.^47^, the matching process (i.e., comparing the current stimulus with the reference) is independent of ‘N’. Thus, theoretically and empirically, the systematic differences in the distance to the referenced WM item among conditions do not confound the computation of behavioral performance in each condition and the computation of gating parameters.

### Procedure

First, participants were welcomed and seated in the EEG lab, the informed consent form was signed, and the EEG cap was prepared. Participants performed the task on a 19-inch computer monitor with a light gray background. Participants were asked to place their left index finger on the left control key of a standard QWERTY keyboard and their right index finger on the right control key. Standardized instructions were given, and a practice run of 60 trials was conducted to familiarize participants with the task. Each block consisted of 60 trials, with participants completing 16 blocks (960 trials in total). A fixation cross was displayed for 800 ms before each trial, and the screen turned blank for 1000 ms. The stimuli X or O were presented with either a red or a blue frame. All blocks started with a reference trial that required no response. The stimuli of each trial (X or O) were randomly selected. The presentation of the target stimulus ended with a keypress, regardless of whether the response was correct or incorrect or when the response was not given within 1400 ms. Participants had to indicate whether the stimulus (X or O) was the exact (match) or different (mismatch) compared to the stimulus that appeared in the last red frame (i.e., the last reference trial). ‘Same’ was indicated by pressing the right control button, whereas ‘different’ was indicated by pressing the left control button with the respective index finger. The response mapping (left/right) was balanced, and the order of correct responses was randomized. In 75% of trials, the type of trial (reference or comparison) was identical to the last trial (nonswitch); 25% of trials were different (switch).

### EEG recordings and pre-processing

The EEG was recorded from 60 equidistant Ag/AgCl electrodes at a sampling rate of 500 Hz using QuickAmp and BrainAmp amplifiers (Brain Products GmbH, Germany). The ground and reference electrodes coordinates were theta = 58, phi = 78 and theta = 90, phi = 90, respectively. After recording, the EEG data were pre-processed using the BrainVision Analyzer 2 software (Brain Products GmbH, Germany). First, the raw data was down-sampled to 256 Hz, and Infinite Impulse Response (IIR) filters with a high-pass of 0.5 Hz, and a low-pass of 40 Hz were applied at a slope of 48 dB/oct. After that, defective channels were discarded, and the remaining channels were re-referenced to an average reference.

Further, a manual raw data inspection was employed to remove technical artifacts, and an independent component analysis was employed to remove regular artifacts like eye movements, blinks, and pulse artifacts. Afterward, the discarded channels were interpolated using a spherical method. After these EEG pre-processing steps, we segmented the continuous data into single trials according to the stimulus onset. The time window of each trial began the 2000 ms before stimulus onset and ended at 2000 ms after stimulus onset. After segmentation, an automatic artifact rejection was processed to remove the residual artifacts, and only trials meeting the following criteria survived: difference of values in an interval of 200 ms is <200 μV; the amplitude is between −200 and 200 μV; the activity in an interval of 100 ms is larger than 0.5 μV. Subsequently, a baseline correction was applied on each trial using a baseline window of −200 ~ 0 ms locked to the stimulus. Baseline-corrected trials without artifacts and with a correct response in 1500 ms after stimulus onset were categorized into four conditions as in the behavioral data.

### Independent component analysis

We used Group-ICA to decompose the EEG data from the four conditions above. In the standard ICA model (applied to single-subject data), different sets of components would be estimated in different orders and scales, and it would be impossible to extract the corresponding components across subjects. Applying Group-ICA addresses this issue. Using the equation C = WX, a Group-ICA tries to extract independent brain sources (C), indicating homogeneous neurophysiological activation of Group EEG data (X) across subjects. W represents the de-mixing matrix. X is the single-trial EEG data because mathematically, for ICA implementation, a certain number of time points are required, and average data does not have sufficient time points. The Group-ICA approach combines data from all subjects’ observations, estimates aggregate components directly, and then reconstructs estimated components back to individual data for group analysis^48^. For this purpose, first, the EEG data are compressed to 1… L factors with principal component analysis to reduce computational load. In the current study, we considered a threshold for the percentage of eigenvalues retaining (98%), and the results showed that by selecting 20 principal components (default number of components in EEGIFT toolbox), the amount of this retaining for all subjects was higher than the threshold. Individual principal components are then concatenated to an aggregate dataset. Afterward, ICA (Infomax method) estimates W using the aggregate components.

This step of Group-ICA estimates the algorithmic and statistical reliability of a group independent component using the ICASSO method^33^. Because the starting point for learning is chosen at random for several ICA algorithms and the sample size is finite, results may vary between runs, even for the same data. To acquire valid estimations of the IC structure, it is thus required to execute the algorithm numerous times with various data distributions and initial values. Bootstrap is a method for changing the distribution of time points across several Group-ICA runs. According to Himberg et al.^33,49^, the ICASSO consists of the following steps:

At first, parameters for the estimation algorithm are selected. These parameters relate to the ICA algorithm and not the ICASSO. The number of components, the number of iterations, and other parameters are examples of this. We used Infomax as an ICA algorithm and the default parameters that were selected in the Group-ICA toolbox. Because the performance of the different algorithms is not remarkably different when applied to aggregate EEG time domain data, changing the algorithm and parameters would not affect the results^50^. After selecting the ICA algorithm and its parameters, the estimation is run *N* times on the bootstrapped data. We applied ICA 50 times on the group independent components estimated for each condition.

Afterward, mutual similarities between all the estimates are computed. As the measure of similarity, we use the absolute value of the linear correlation coefficient between the independent components. The estimates are clustered according to their mutual (dis)similarities. In principle, the clustering method can be freely selected. We applied the default agglomerative hierarchical method of the Group-ICA toolbox for clustering. According to Himberg et al.^33^, the most reasonable initial number of ICASSO clusters would equal the data dimension n. Since we had applied principle component analysis on the data before ICASSO and we had extracted 20 principle components, we selected 20 for the number of final ICASSO clusters. We computed the R-index to mathematically further validate that the number of final clusters was optimal. The R-index approach is used to select the number of disjoint ICASSO clusters^51^. It is a quantitative measure that can be computed knowing only the similarity matrix. The algorithm to calculate the R-index searches for compact and well-separated clusters and the minimum of it suggests the best partition. The lowest number of the R-index shows the best clustering qualification for the data.

The clustering can be visualized as a 2D plot or as a dendrogram. In the current study, we visualized clusters as a similarity 2D plot to have a better visualization of the cluster compactness. The clustering of the estimates is expected to reveal information about the estimation’s reliability (robustness). The compactness of the clusters shows their reliabilities. A compact cluster develops when a similar estimate appears repeatedly despite the randomization. Also, for each ICASSO cluster, an index (*I*_q_) was defined to show its compactness. *I*_q_ was calculated as a difference between intra and inter-cluster components’ similarity^49^. By defining a threshold for *I*_q_, compact clusters with cluster indices higher than the threshold correspond to reliable estimations. The *I*_q_ threshold for defining reliable components in the current investigation was 0.85^52^. Finally, each cluster is represented by a single ICA estimate, with the IC representing the highest number of similarities to other cluster points.

Thus, the parameter choice in the ICASSO was clearly constrained by mathematical procedures. After that, for the ICs representing compact clusters, the Group-ICA algorithm performed a back-reconstruction to retrieve individual components. We used the EEGIFT toolbox to implement the Group-ICA application (http://icatb.sourceforge.net/.EEGIFT).

After extracting group components for gate opening and closing conditions, we applied a clustering technique to find spatially similar components between two task conditions. CORRMAP^53^ is a clustering method that classifies ICs similar to one or a few templates (http://www.debener.de/corrmap). The component similarity is measured using a correlation procedure that selects components that pass a defined threshold. The template is compared with all component maps from all datasets by calculating a correlation value. All components with an absolute correlation that equals to or greater than the threshold are selected to be part of the cluster. CORRMAP is just a clustering method and can be used for finding spatially similar brain-related sources. From a mathematical point of view, it is the similarity of data distributions that is estimated, and it does not matter whether an artifact template is used for comparison (the initial implementation of CORRMAP) or whether a cognitive IC is used. Each of the reliable components of two different conditions (based on the ICASSO results) was considered as the template of each CORRMAP cluster separately, and the spatially similar ICs from all conditions with a correlation coefficient higher than the threshold were classified. Because the CORRMAP clustering only depends on the selected template and not its origin (i.e., artifact or brain-related component), we used brain-related sources to extract similar cognitive ICs among different conditions. We considered the threshold of correlation between independent component topographies to be 0.9. For each template, we set a maximum of three representing components of each group to find the most similar components. This prevented CORRMAP clustering from finding similar overlapping components.

### Multivariate pattern analysis

Two MVPA analyses were executed at the single-subject level for each IC pairs reflecting gate opening and closing using the MVPA-light toolbox^54^: a binary classification across time and a temporal generalization analysis. For each trial, only signals from 0 to 1500 ms after stimulus onset were fed into the MVPA as we only considered the post-stimulus processes. Before the MVPA for each participant and each IC pair was conducted, under-sampling was performed to balance the number of trials in two classes to avoid an overfitting problem. The removed trials during under-sampling processes were randomly selected.

Binary classification and temporal generalization analyses were done separately for each individual and IC pair using the same classifier and parameter settings. We used the default linear discriminant analysis classifier in the MVPA-Light toolbox to contrast two conditions. Since the signal-noise ratio increases after ICA, we used this more time-efficient approach instead of a suggested support vector machine method. A five-fold cross-validation method was applied twice for binary classification and temporal generalization. Specifically, the dataset is randomly split into 5 folds in each cross-validation process. In each iteration step, one fold was used as the testing dataset and the rest as the training dataset. Cross-validation was repeated once. The final performance of cross-validation was averaged from the two repetitions.

Cluster-based permutation tests were run on both MVPA performances represented by the AUC values to identify the time points showing significant classification performance. All cluster-based permutation tests were based on the non-parametric Wilcoxon tests on each time point (for binary classification across time and time generalization). The null value for AUC was at a chance level of 0.5. The reference distribution of the permutation test was computed with 1000 random draws. The threshold for the Wilcoxon tests was 0.05. The cluster-level statistics were computed using the sum of all Wilcoxon test values within time points.

### Source localization analysis

To examine the functional neuroanatomical sources associated with gate opening and gate closing sub-processes identified by different IC pairs, sLORETA (standardized low-resolution brain electromagnetic tomography) was conducted^35^ on back-projected data only for the IC pairs showing significant temporary generalization performance in MVPA process. sLORETA was conducted to extract the differential modulations for each IC pair between gate opening and gate closing conditions. The time points selected for the ICs accord to the temporary generalization results in the MVPA process. There is converging evidence from EEG/(f)MRI and EEG/TMS studies that the source provided by sLORETA is reliable^55–57^. sLORETA has also been shown to be usable in IC-decomposed EEG data and when interested in the sources of different ICs^58^. sLORETA partitions the intracerebral volume into 6239 voxels (using the MNI152 template), resulting in a spatial resolution of 5 mm. The basis of this partitioning step is a realistic head model. For each of the voxels, the standardized current density is calculated. According to the theoretical framework underlying the design of the reference back paradigm, gate opening processes can be calculated by contrasting reference_switch and reference_nonswitch trials. Gate closing processes can be calculated by contrasting comparison_switch with comparison_nonswitch trials. These different contrasts were calculated for the time period showing temporal generalization in the MVPA analysis. For the statistical comparisons, the voxel-based sLORETA images for the different contrasts were calculated using the sLORETA-built-in voxel-wise randomization tests based on statistical non-parametric mapping (SnPM) (2500 permutations were used). Voxels with significant differences (*p* < 0.05, corrected for multiple comparisons) between contrasted conditions were located in the MNI-brain (www.unizh.ch/keyinst/NewLORETA/sLORETA/sLORETA.htm). EEG source reconstruction is limited in its spatial resolution and the findings reported may require validation by functional imaging. Yet, in the context of the current study in which we are interested in how the temporal process of neural activity evolves across different brain structures, EEG source localization is the best way to go, because the time resolution of EEG is combined with a reasonable spatial resolution of this methods.

### Statistics and reproducibility

We used IBM SPSS Statistics 28.0.1.1 for the analysis of the behavioral data with a sample size of *N* = 33. The mean and standard deviation are given for the descriptive statistics. Mean RTs and mean accuracy (percentage of correct responses) were analyzed for every participant and each condition to calculate gate closing and gate opening. Only trials with correct responses were analyzed. According to Kolmogorov–Smirnov tests, all behavioral variables included in the analyses were normally distributed (all *p* > 0.120). A paired sample *t*-test was used to analyze the differences between gate opening and gate closing for the behavioral data. The sample size is larger than previous studies using the same experimental setup^11^ and similar to recent studies using EEG-based MVPA^25–27^. All information about the statistical comparison can be found in the above sections and the results. All custom analysis codes have been deposited in OSF.

### Reporting summary

Further information on research design is available in the Nature Research Reporting Summary linked to this article.

## Supplementary information

Supplementary Information

Reporting Summary

## Data Availability

Source data underlying figures can be downloaded from https://osf.io/jw6v2/^59^.

## Code availability

Custom code used to process the data can be found at https://osf.io/jw6v2/^59^.
