# Supplementary material for: A ventral stream-prefrontal cortex processing cascade enables working memory gating dynamics
Source: Commun Biol. 2022 Oct 12;5:1086. doi: 10.1038/s42003-022-04048-7 (PMC9556714; doi:10.1038/s42003-022-04048-7)
Supplement: Supplementary file 1 — Supplementary Information [file 42003_2022_4048_MOESM1_ESM.pdf]

Supplementary Information

A ventral stream-prefrontal cortex processing cascade enables working memory gating dynamics

Shijing Yu, Sarah Rempel, Negin Gholamipourbarogh, Christian Beste

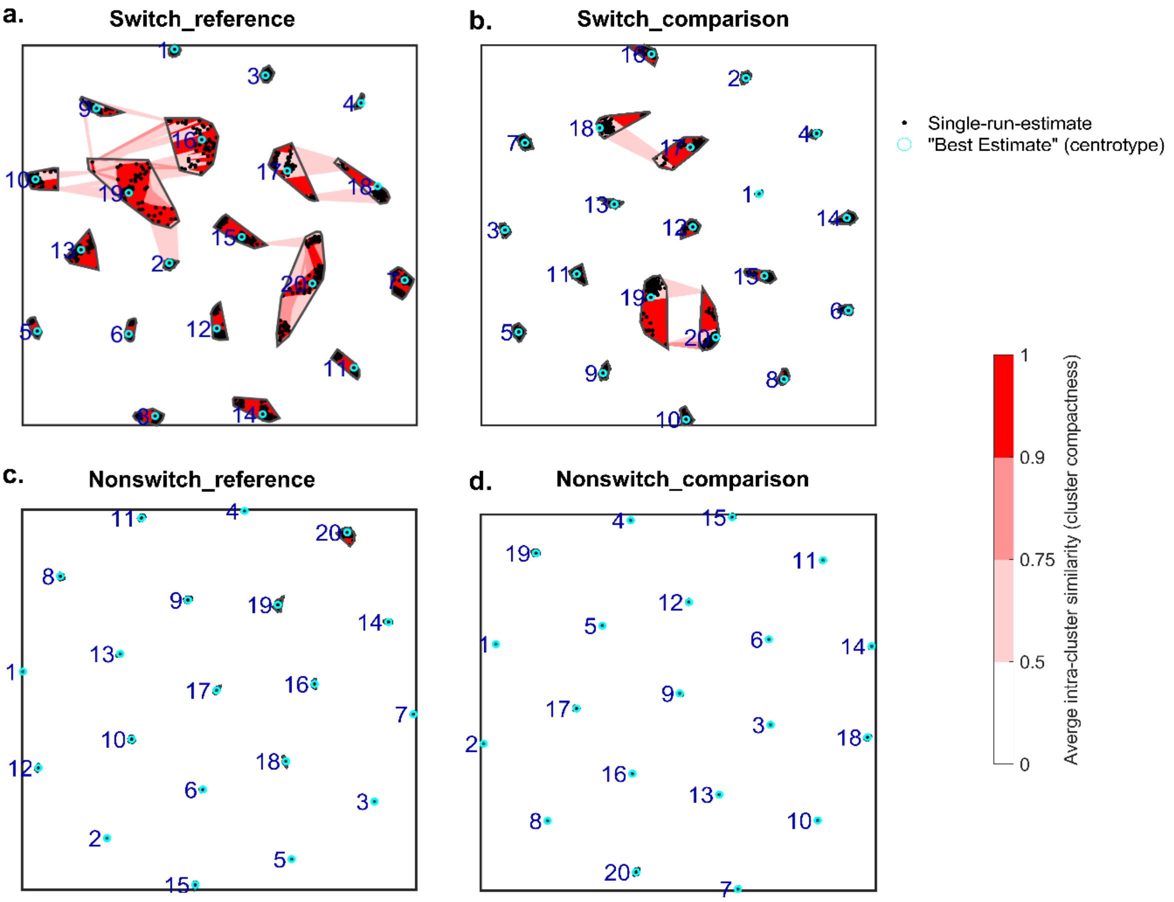

Supplementary Figure 1. ICASSO cluster maps for all four conditions showing the reliability of the ICA decomposition. ICASSO is a sub process of Group ICA for finding reliable group components. ICA is applied 60 times and the extracted components are clustered based on their mutual similarity. Clusters are visualized as a 2D plot and the compactness of each cluster shows the reliability of the corresponding component. These cluster maps are completely different form CORRMAP clustering results. Convex hulls represent estimate-clusters. Compact and isolated clusters suggest reliable estimates. Note that the pairwise similarity graph between estimates inside clusters is omitted if the average intra-cluster similarity is above 0.90.

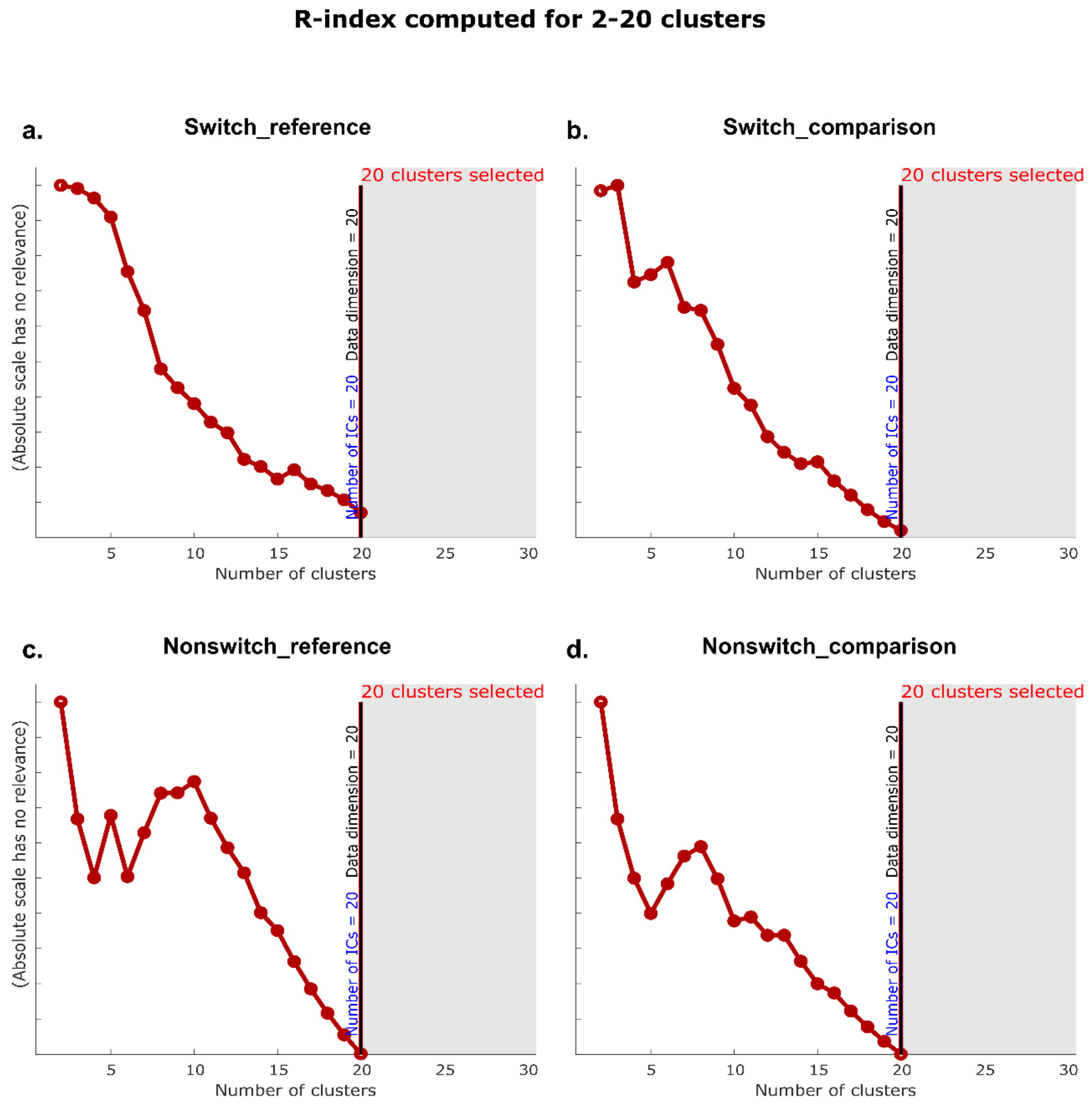

Supplementary Figure 2 The R-index values for the number of clusters in ICASSO algorithm for different conditions. The lowest value of the Y axis shows the best quality of the clustering. For all conditions, 20 (equals to the number of components) was the best choice for the cluster size.

Supplementary Table 1. Ppaf for similar IC pairs extracted from CORRMAP clustering for the gate opening conditions

Pair index		1	2	3	4	5	6	sum
Ppaf (IC index)	Switch reference	3.41% (IC 2)	3.10% (IC 5)	87.73% (IC 7)	2.31% (IC 8)	1.97% (IC 11)	6.17% (IC 18)	96.29%
	Nonswitch reference	0.83% (IC 18)	0.69% (IC 6)	83.93% (IC 8)	1.55% (IC 20)	3.49% (IC 14)	1.06% (IC 5)	89.93%

Supplementary Table 2. Ppaf for similar IC pairs extracted from CORRMAP clustering for the gate closing conditions

Pair index	1	2	3	4	5	sum

Ppaf (IC index)	Switch comparison	51.88% (IC 7)	3.85% (IC 8)	3.04% (IC 15)	28.14% (IC 19)	5.93% (IC 20)	89.42%
	Nonswitch comparison	81.73% (IC 15)	0.16% (IC 6)	0.37% (IC 12)	18.53% (IC 19)	1.79% (IC 17)	98.84%

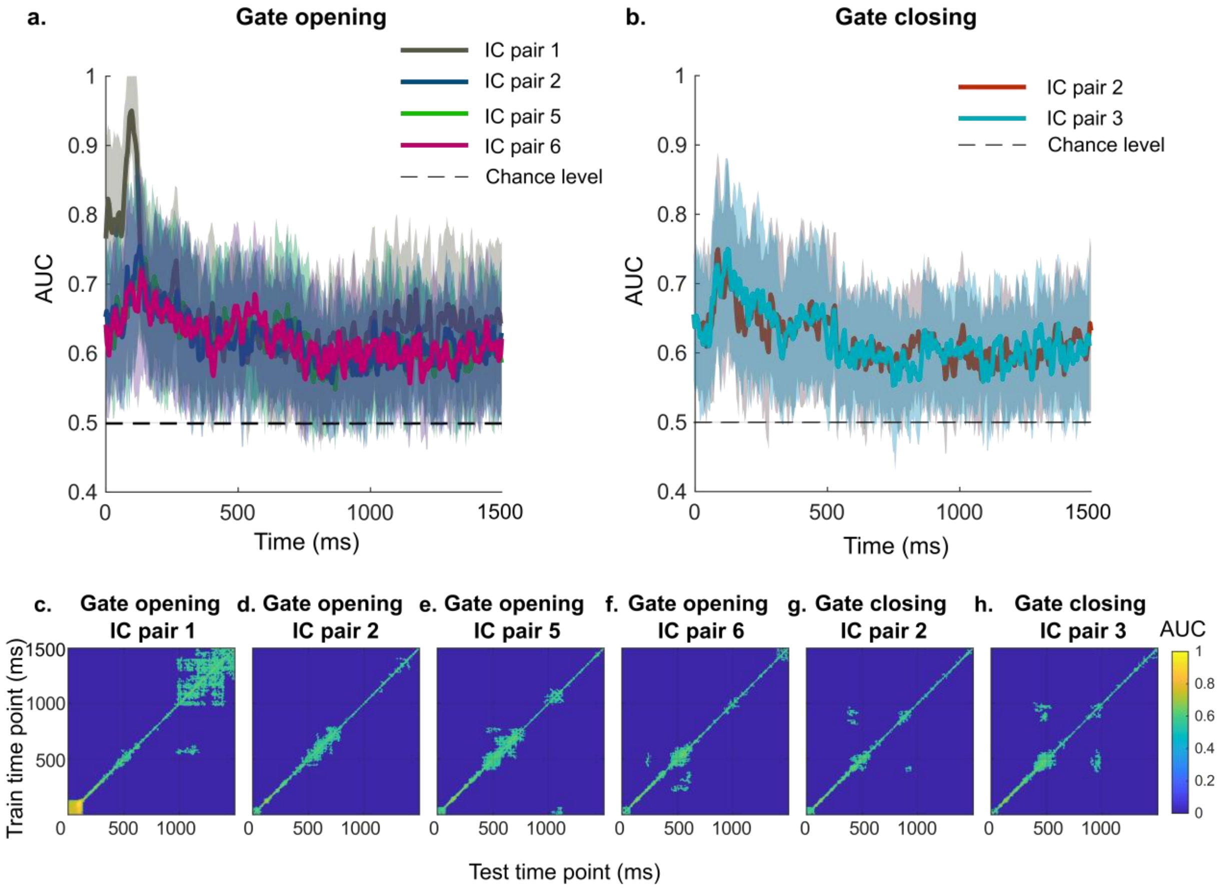

Supplementary Figure 3 MVPA results for IC pairs with non-evident temporal generalization clusters. Plots (a) and (b) show the binary classification performance of gate opening and closing conditions separately. The shaded error bars represent standard deviation. Plots (c) to (h) illustrate the temporary generalization of each IC pair. Only time points with significant ($p<.05$) classification performance computed by cluster-based permutation test with N = 33 samples are presented in color.
