# Supplementary material for: A ventral stream-prefrontal cortex processing cascade enables working memory gating dynamics
Source: Commun Biol. 2022 Oct 12;5:1086. doi: 10.1038/s42003-022-04048-7 (PMC9556714; doi:10.1038/s42003-022-04048-7)
Supplement: Supplementary file 2 — Reporting Summary [file 42003_2022_4048_MOESM2_ESM.pdf]

nature portfolio

Corresponding author(s): Christian Beste

Last updated by author(s): Mar 25, 2022

Reporting Summary

Nature Portfolio wishes to improve the reproducibility of the work that we publish. This form provides structure for consistency and transparency in reporting. For further information on Nature Portfolio policies, see our Editorial Policies and the Editorial Policy Checklist.

Statistics

For all statistical analyses, confirm that the following items are present in the figure legend, table legend, main text, or Methods section.

n/a	Confirmed	
☐	☒	The exact sample size (*n*) for each experimental group/condition, given as a discrete number and unit of measurement
☐	☒	A statement on whether measurements were taken from distinct samples or whether the same sample was measured repeatedly
☐	☒	The statistical test(s) used AND whether they are one- or two-sided<br>*Only common tests should be described solely by name; describe more complex techniques in the Methods section.*
☒	☐	A description of all covariates tested
☐	☒	A description of any assumptions or corrections, such as tests of normality and adjustment for multiple comparisons
☐	☒	A full description of the statistical parameters including central tendency (e.g. means) or other basic estimates (e.g. regression coefficient) AND variation (e.g. standard deviation) or associated estimates of uncertainty (e.g. confidence intervals)
☐	☒	For null hypothesis testing, the test statistic (e.g. *F*, *t*, *r*) with confidence intervals, effect sizes, degrees of freedom and *P* value noted<br>*Give P values as exact values whenever suitable.*
☐	☒	For Bayesian analysis, information on the choice of priors and Markov chain Monte Carlo settings
☒	☐	For hierarchical and complex designs, identification of the appropriate level for tests and full reporting of outcomes
☐	☒	Estimates of effect sizes (e.g. Cohen's *d*, Pearson's *r*), indicating how they were calculated

Our web collection on statistics for biologists contains articles on many of the points above.

Software and code

Policy information about availability of computer code

Data collection	EEG recording: BrainVision Recorder (Brain Products GmbH)<br>Stimulus Presentation: Presentation 14.9 (Neurobehavioral Systems, Inc.)
Data analysis	Statistics behavioral data: IBM SPSS Statistics (IBM)<br>EEG processing: BrainVision Analyzer 2 (Brain Products GmbH); Matlab (The MathWorks, Inc.)<br>EEGIFT toolbox (http://icatb.sourceforge.net/.EEGIFT)<br>MVPA light toolbox<br>Source localization: sLORETA (Pascual-Marqui, KEY Institute for Brain-Mind Research, University Hospital of Psychiatry Zurich; http://www.unizh.ch/keyinst/NewLORETA/sLORETA/sLORETA.htm

For manuscripts utilizing custom algorithms or software that are central to the research but not yet described in published literature, software must be made available to editors and reviewers. We strongly encourage code deposition in a community repository (e.g. GitHub). See the Nature Portfolio guidelines for submitting code & software for further information.

Data

Policy information about availability of data

All manuscripts must include a data availability statement. This statement should provide the following information, where applicable:

- Accession codes, unique identifiers, or web links for publicly available datasets
- A description of any restrictions on data availability
- For clinical datasets or third party data, please ensure that the statement adheres to our policy

Data can be downloaded from https://osf.io/6n7uc

Field-specific reporting

Please select the one below that is the best fit for your research. If you are not sure, read the appropriate sections before making your selection.

☐ Life sciences ☒ Behavioural & social sciences ☐ Ecological, evolutionary & environmental sciences

For a reference copy of the document with all sections, see nature.com/documents/nr-reporting-summary-flat.pdf

Behavioural & social sciences study design

All studies must disclose on these points even when the disclosure is negative.

Study description	quantitative, within-subject design of experimental conditions
Research sample	student sample from TU Dresden
Sampling strategy	convenience sample
Data collection	EEG data and behavioral data (computer keyboard responses during an experiment)
Timing	January 2020 until October 2020
Data exclusions	N=9 as stated in the main text
Non-participation	there were no drop-outs
Randomization	no randomization, it is a within-subject design

Reporting for specific materials, systems and methods

We require information from authors about some types of materials, experimental systems and methods used in many studies. Here, indicate whether each material, system or method listed is relevant to your study. If you are not sure if a list item applies to your research, read the appropriate section before selecting a response.

Materials & experimental systems

n/a	Involved in the study	
☒	☐	Antibodies
☒	☐	Eukaryotic cell lines
☒	☐	Palaeontology and archaeology
☒	☐	Animals and other organisms
☐	☒	Human research participants
☒	☐	Clinical data
☒	☐	Dual use research of concern

Methods

n/a	Involved in the study	
☒	☐	ChIP-seq
☒	☐	Flow cytometry
☒	☐	MRI-based neuroimaging

Human research participants

Policy information about studies involving human research participants

Population characteristics	Data of Rempel et al. [2021] (N=24 individuals) and an unpublished dataset (N=18 individuals) were chosen for this study (N=42 individuals in total; 13 males, mean age 25.62 ± 5, all right-handers). To ensure a high quality of the group ICA analysis, we excluded N=9 participants with an insufficient trial number (< 50) for each condition after EEG segmentation. The final

	sample comprised N=33 participants (9 males, mean age: 25.77 ± 5.01). This sample is comparable to other studies examining representational dynamics of neurophysiological activity using MVPA .
Recruitment	convenience sample, participants were recruited via volunteers board and panel advertisements
Ethics oversight	IRB of the TU Dresden a

Note that full information on the approval of the study protocol must also be provided in the manuscript.
